# Auditory dysfunction in type 2 Stickler Syndrome

**DOI:** 10.1007/s00405-020-06306-y

**Published:** 2020-09-08

**Authors:** Philip Alexander, Philip Gomersall, Jack Stancel-Lewis, Gregory Scott Fincham, Arabella Poulson, Allan Richards, Annie McNinch, David M. Baguley, Martin Snead

**Affiliations:** 1 Vitreoretinal Service, Box 41, Addenbrooke’s Hospital, Cambridge University Hospitals NHS Foundation Trust, Hills Road, Cambridge, CB2 0QQ UK; 2 NHS England Stickler Syndrome Diagnostic Service, Box 153, Cambridge University NHS Foundation Trust, Hills Road, Cambridge, CB2 0QQ UK; 3 Ear, Nose and Throat (ENT) West Wing, Oxford Auditory Implant Programme, John Radcliffe Hospital, Oxford, OX3 9DU UK; 4 NHS England and NHS Improvement, Wellington House 33-155 Waterloo Road, London, SE1 8UG UK; 5 Department of Pathology, University of Cambridge, Cambridge, CB2 1QP UK; 6 Regional Molecular Genetics Laboratory, Addenbrooke’s Hospital, Cambridge University Hospitals NHS Foundation Trust, Hills Road, Cambridge, CB2 0QQ UK; 7 National Institute for Health Research (NIHR) Nottingham Biomedical Research Centre, Nottingham, NG1 5DU UK; 8 Hearing Sciences, Division of Clinical Neuroscience, School of Medicine, University of Nottingham, Nottingham, NG7 2UH UK; 9 Nottingham Audiology Services, Nottingham University NHS Trust, Nottingham, NG1 3DU UK; 10 Nottingham Biomedical Research Centre, Ropewalk House, 113 Ropewalk, Nottingham, NG1 5DU UK

**Keywords:** Stickler Syndrome, Type 2, Conductive, Sensorineural, Hearing loss, Retinal detachment, COL11A1, COL2A1

## Abstract

**Purpose:**

To present the extent and site of lesion of auditory dysfunction in a large cohort of individuals with type 2 Stickler Syndrome. Type 2 Stickler Syndrome results from a mutation in the gene coding for α-1 type XI pro-collagen, which has been identified in the human vitreous, cartilage and the cochlea of the mouse. The condition is characterised by classic ocular abnormalities, auditory dysfunction, osteoarthropathy and oro-facial dysplasia.

**Methods:**

This is a population study which used a combination of audiometric, tympanometric, and self-report measures on a series of 65 individuals (mean age 29.2 years, range 3–70, female 63.1%) with genetically confirmed type 2 Stickler Syndrome.

**Results:**

Hearing impairment was identified in at least one ear for 69% of individuals. Analysis against age-matched normative data showed that reduced hearing sensitivity was present across all test frequencies. Sensorineural hearing loss was most common (77% of ears), with conductive (3%), mixed (7%) and no hearing loss (13%), respectively. The proportion of hypermobile tympanic membranes (24%) was less than previously documented in type 1 Stickler Syndrome. When present, this appears to arise as a direct result of collagen abnormalities in the middle ear. Self-report measures of speech and spatial hearing in sound were comparable to a non-syndromic cohort with similar audiometric thresholds.

**Conclusions:**

Auditory impairment in type 2 Stickler Syndrome is predominantly associated with cochlear hearing loss of varying severities across affected individuals. The impact on hearing thresholds can be seen across the frequency range, suggesting a contribution of defective collagen throughout the cochlea. Self-report questionnaires showed that difficulties understanding speech, and spatial information in sound (such as that used for localisation), were worse than a young, normal-hearing population but comparable to a non-syndromic cohort with similar audiometric thresholds. Therefore, it is likely that hearing loss in type 2 Stickler Syndrome arises in the auditory periphery, without significant central processing deficits.

## Introduction

In 1965, Dr. Gunnar Stickler and colleagues described a dominant “hereditary arthro-ophthalmopathy” characterised by premature degenerative osteoarthropathy, and progressive myopia beginning in the first decade of life and frequently resulting in retinal detachment and blindness [1]. Originally considered a single gene disorder, at least nine genes are now known to cause Stickler Syndrome [2, 3], but the vast majority result from abnormalities in either Type II, Type IX or Type XI collagen. This cluster of conditions, best referred to as the Stickler Syndromes, is among the most frequently inherited connective tissue disorders, accounting for 1:7500 births [4, 5]. Type 1 Stickler Syndrome, caused by mutations in COL2A1 (the gene encoding the α-1 chain for Type II collagen) accounts for more than 80% of Stickler Syndrome patients [6]. Type 2 Stickler Syndrome results from a mutation in the gene coding for α-1 type XI pro-collagen, which has been identified in the human vitreous, cartilage and the cochlea of the mouse. The condition is characterised by classic ocular abnormalities, auditory dysfunction, osteoarthropathy and oro-facial dysplasia.

Mild hearing loss, affecting the mid- to high frequencies, was first associated with this condition by Stickler and Pugh in 1967 [7]. Auditory dysfunction in the Stickler Syndromes may be conductive, sensorineural or mixed. Conductive hearing loss secondary to glue ear and serous otitis media is a well-recognised complication of cleft palate [8, 9], which itself is a feature of the Stickler Syndromes; ossicular abnormalities may also contribute to conductive loss [10]. Sensorineural hearing loss can also occur but its pathogenesis is less certain [11].

Hearing loss is thought to be more common and more severe in type 2 Stickler Syndrome, caused by mutations in COL11A1, than in type 1 Stickler syndrome. In the first description of the Stickler Syndrome associated with a COL11A1 mutation, Richards et al. [12], reported sensorineural hearing loss in 6 out of 7 individuals of a single family. Poulson et al. [13] described 31 patients, from 6 pedigrees, with genetically confirmed type 2 Stickler Syndrome and the typical beaded vitreous phenotype. Of these, only 45% reported hearing difficulties, but 80% of those tested had mild or moderate high-frequency sensorineural hearing loss demonstrated on audiometry, with only a small proportion (10%) having superimposed conductive components (mixed hearing loss) [13].

Relatively few studies have described the auditory phenotype of the Stickler Syndromes in detail. Acke et al. [14] conducted a meta-analysis of the auditory phenotype of 313 patients with Stickler Syndrome. Sixty-three percent of all patients were reported to have hearing loss, with the prevalence of hearing loss being 52% in type 1, compared to 82.5% in type 2. In both groups, the majority of patients had sensorineural or mixed hearing loss, with only a minority (10.4% and 5.3% in type 1 and type 2, respectively) showing pure conductive loss.

Szymko-Bennett and colleagues assessed auditory dysfunction by means of audiometry and tympanometry in 46 Stickler Syndrome patients from 26 family pedigrees [15]. When hearing loss was categorised into frequency and severity categories, the authors noted a greater severity in the high frequencies and in the older age group, with a prominence of mild-to-moderate impairment. However, when thresholds were adjusted for age-dependent normative data the dependence of the hearing loss on frequency was less obvious. 60% of individuals had at least two thresholds outside the 95th percentile of their age-matched reference data. Linear regression of hearing threshold at 4 kHz, 6 kHz and 8 kHz thresholds showed no significant progression of hearing loss beyond that expected of ageing (presbyacusis). Tympanometry assessed in 46 ears demonstrated that 21 (46%) were hypermobile, a higher proportion than would be expected in a normal population. Genetic clarification was not sought in this study, although the authors presumed that “most, if not all” subjects had genetic abnormalities in COL2A1 (type 1 Stickler Syndrome).

Hearing assessment of only a limited number of individuals with type 2 Stickler Syndrome have been reported in the literature, which motivates the need to study and report a larger population. Furthermore, there is a lack of information guiding how classifications of severity and aetiology should be made, and much variation in reporting, where this information is available in the Stickler Syndromes. Consequently, to establish the relative contribution of type 2 Stickler Syndrome on hearing impairment, in the context of the expected role of ageing of the auditory system (presbyacusis), careful comparison of findings with age- and gender-matched normative data is required.

The aim of the present study was to examine the auditory phenotype of individuals with genetically confirmed type 2 Stickler Syndrome. We aimed to compare the relative proportions of conductive/mixed and sensorineural losses, and re-examine reports that hearing loss in type 2 Stickler Syndrome is restricted to high frequencies by comparing hearing thresholds with age- and gender-matched normative data. Tympanometry was used to determine the proportion of individuals with hypermobile tympanic membranes to facilitate comparison with previous studies. To ascertain if tympanic membrane hypermobility is acquired through repeated infection-related trauma, a clinical questionnaire regarding previous history of ear infections and otological symptoms was collected. In addition, results of a clinically validated hearing loss questionnaire, are reported and compared to data from a population with similar audiometric profiles due to ‘typical’ aetiologies that contribute to hearing loss. If hearing difficulties reported were significantly greater in the Stickler population than the comparative population, this would suggest a potential role of central auditory processing alongside peripheral auditory impairment.

This is the largest cohort of genetically confirmed type 2 Stickler Syndrome patients in the literature, and the only report detailing the auditory phenotype in this sub-group of the disorder.

## Methods

Subjects with genetically confirmed type 2 Stickler Syndrome were recruited via the Nationally Commissioned Highly Specialised Stickler Syndrome diagnostic service held at Cambridge University Hospitals, UK (CUH). Data collected on each patient included demographics, otological history, pure-tone audiometry (PTA), tympanometry, and clinically validated auditory handicap questionnaires. This project was conducted following National Research Ethics Service approval (10/H0301/57).

### Otological history

Past otological history was assessed using a bespoke patient questionnaire (see appendix 2.1). The questionnaire documented subjective accounts of ear-related pathology, including self-reported hearing loss, frequency of ear infections, history of otological surgery and potential otological symptoms associated with auditory dysfunction.

### Pure-tone audiometry

Pure-tone audiometry was undertaken in a sound proofed booth following the Recommended Procedure of the British Society of Audiology [16] utilising a calibrated Madsen Astera audiometer. Hearing impairment was defined as a pure-tone four-frequency average (4-FA) > 20 decibels Hearing Level (dBHL) in at least one ear. The 4-FA was calculated as the average of pure-tone thresholds at 500, 1000, 2000 and 4000 Hz. This definition of hearing impairment is relatively conservative, but is selected to best represent a hearing impairment associated with significant impact on the individual’s quality of life. Hearing impairment severity is calculated from the better ear four-frequency average. The degree of hearing loss was defined using British Society of Audiology (BSA) criteria for PTA (mild hearing loss 20–40 dBHL; moderate hearing loss 41–70 dBHL; severe hearing loss 71–95 dB HL; profound hearing > 95 dB HL) [17]. Conductive hearing loss was defined as an air–bone gap > 20dBHL at two or more test frequencies between 1 and 4 kHz. The diagnosis of mixed hearing loss was made if the audiograms met the conductive hearing loss criteria, but with a bone conduction (BC) threshold > 20 dBHL at 500 Hz, 1 kHz, 2 kHz or 4 kHz. Data from sound field audiometry, and any tests, where pure-tone thresholds at 500 Hz, 1 kHz, 2 kHz and 4 kHz were not present, in addition to any test in which BC thresholds had not been sufficiently masked, were excluded from analysis.

### Tympanometry

Objective tympanometry (probe tone 226 Hz) assessed middle ear function by evaluating the impedance of sound by the tympanic membrane and middle ear structures [18]. A calibrated GSI Tympstar device was utilised. Tympanometry was performed and results assessed according to BSA guidelines [19], in addition to the Jerger classification method [20] (see Table 2). Tympanograms with a peak middle ear admittance  > 1.6 cm^3^ were considered as hypermobile and labelled as A_d_[18], and normal tympanograms were labelled as type A [18].

### Auditory handicap questionnaire

Self-reported auditory handicap was assessed using the Speech, Spatial and Qualities of Hearing Scale (SSQ) questionnaire [21]. This clinically validated questionnaire contains three sections, each relating to a particular aspect of hearing: speech comprehension, spatial hearing (the ability to locate, judge distance and direction of sounds in the environment), and the clarity and naturalness of sounds. It is particularly sensitive to assessing unilateral hearing loss. Each question was scored by the patient on a continuous scale from 0 to 10 (or non-applicable).

### Statistical methods

Audiograms were independently assessed by two audiologists to classify aetiology in each ear. In addition to classification of severity, audiometric thresholds were compared to an age- and gender-matched normal-hearing population. Only subjects classified as having a purely sensorineural hearing loss were included for this analysis. The proportion of individuals outside the 95^th^ percentile for age- and gender-matched normative data (ISO 7029:2000(E)) was determined at each test frequency for those aged > 18 years. A *χ*^2^ analysis was used to determine if the proportion of individuals falling outside the 95th percentile was significant. Cochran’s *q* test was used to investigate the effect of audiometric test frequency on the proportion of individuals falling outside the normal range.

Tympanometry data was assessed with regard to data reported in individual otological symptom questionnaires. A McNemar analysis was performed to determine if there was a significant difference in aetiology between the individuals presenting with A_d_ tympanogram and those with type A.

Average total SSQ scores from our type 2 Stickler sample were compared to a population with a similarly matched level of hearing impairment used by Gatehouse and Noble [21] when validating the SSQ. An independent samples *t* test was used to compare the means of the two groups to determine if the type 2 Stickler population reported greater hearing difficulty compared to a population of individuals with similar audiometric thresholds arising from other, more typical, causes. *P* values < 0.05 were regarded as statistically significant.

## Results

Eighty-three patients with genetically confirmed type 2 Stickler Syndrome were recruited for study; 65 subjects were included in the analysis after 18 individuals were excluded due to incomplete audiograms. The mean age was 29.02 (range 3–70, SD 19.24). Of the patient group 63.1% were female (*n* = 41 mean age 27.12 years, range 3–64, SD 19.23), and 36.9% male (*n* = 24, mean age 32.25 years, range 5–70, SD 19.23).

### Hearing loss severity

Using the definition of a hearing loss as a four-frequency average (4FA) worse than 20 dB HL, 69% of individuals [45/65] had a hearing loss in at least one ear. A mild hearing loss in the better ear was the most common presentation, affecting 46% [30/65] of the population. The majority of hearing loss was bilateral and symmetrical. The proportion of individuals with different configurations of hearing loss is shown in more detail in Table 1.

**Table 1:** Laterality and severity of hearing impairment (pure-tone audiometry 4-frequency average worse than 20 dBHL in an ear), categorised according to age

Age	M:F	Hearing loss ( 4-FA > 20 dB HL)			Severity (better ear 4-FA)			
		Bilateral (Asymmetric*)	Unilateral	No Hearing Loss	Severe-Profound	Moderate	Mild	Normal
0–19	8:17	14 (0)	1	10	1	3	10	11
20–39	10:14	15 (1)	3	6	1	2	12	9
40–59	4:6	5 (0)	2	3	0	2	3	5
60–80	3:3	5 (1)	0	1	0	0	5	1
All	25:40	39 (2)	6	20	2	7	30	26

Severity is based on British Society of Audiology descriptors applied to the better hearing ear [17]. *Asymmetry is calculated according to the criteria of > 15 dBHL difference between air-conduction thresholds

### Hearing impairment aetiology

**Fig. 1:**
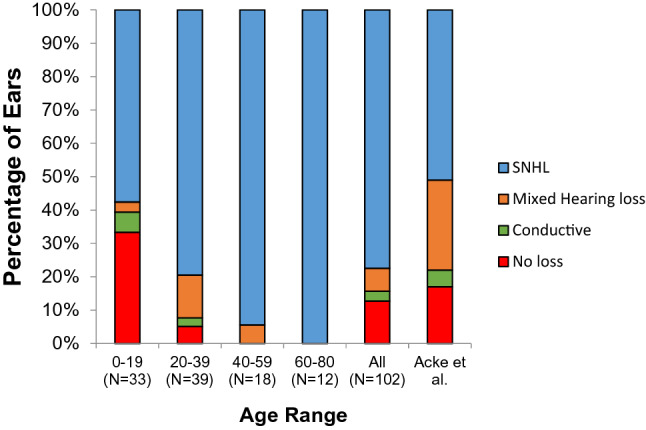
Aetiology of hearing loss in type 2 Stickler Syndrome assessed on PTA and categorised by age. The final column shows meta-analysis aetiology of hearing loss data in both type 1 and 2 Stickler Syndrome published in Acke et al. [16]

The aetiology of hearing loss was assessed by PTA on an ear-by-ear basis. A total of 102 ears of 65 patients were included, following exclusion of ears, where underlying aetiology could not be reliably established. The distribution of aetiologies, categorised according to age is shown in Fig. 1, and compared to previous meta-analysis data [14]. It can be seen that sensorineural hearing loss is the most common type of hearing loss seen in all age groups, affecting 77% of the total number of ears for which audiometric information was obtained. Smaller proportions of individuals presented with conductive (3%) and mixed (7%) hearing losses. Same-day tympanometric information existed for four ears, where a conductive hearing loss had been identified. A type B tympanogram was observed in one case, suggesting a temporary conductive component associated with middle ear effusion) and type A tympanograms were present in the other cases, suggesting a permanent conductive hearing loss.

**Fig. 2:**
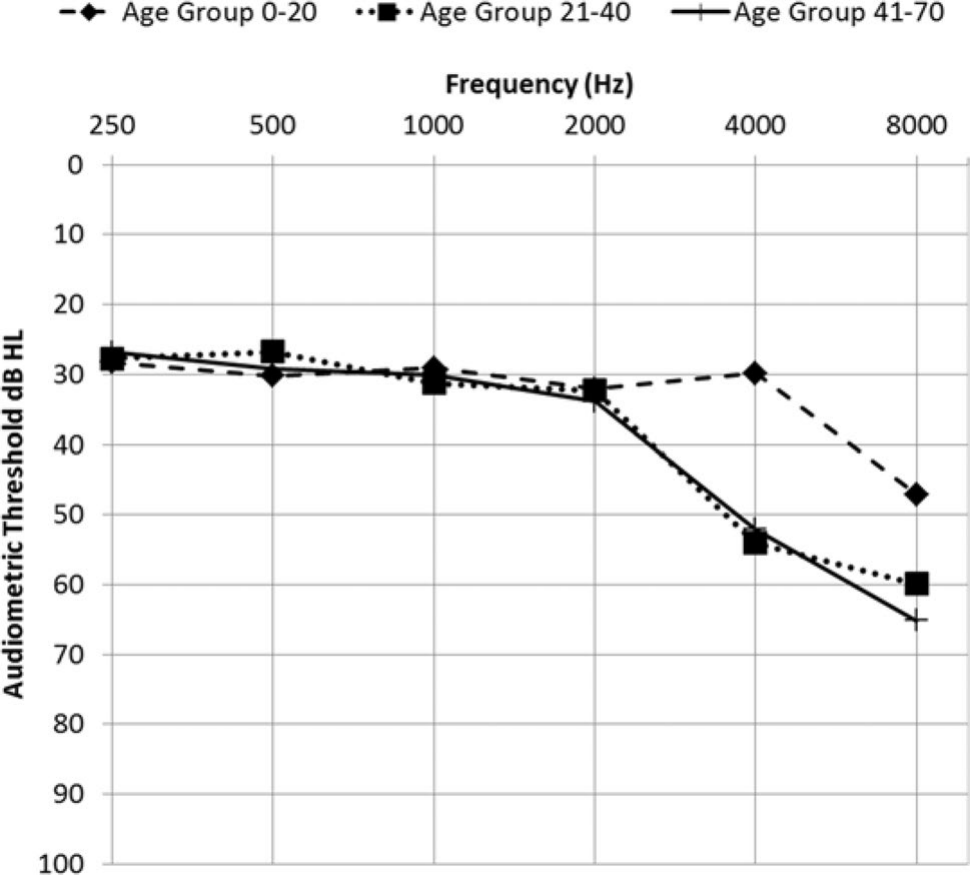
Audiogram of averaged hearing thresholds for three different age groups of patients with type 2 Stickler Syndrome

Averaged hearing thresholds, presented as an audiogram for three different age groups of patients with type 2 Stickler Syndrome, are shown in Fig. 2.

#### Comparison with age-matched normative data

The proportion of ears with sensorineural hearing loss identified to have thresholds falling outside the 95th percentile for each test frequency using age-specific audiometric data, along with corresponding *χ*^2^ and *p* values, is shown in Table 2. It can be seen that a large proportion of the type 2 Stickler cohort have audiometric test thresholds significantly raised compared to those of age-matched normative data. Statistical tests (Chi-squared) were performed to assess whether there was a relationship between raised hearing thresholds and test frequency. A significant influence of audiometric test frequency on the proportion of values that fall outside the 95th percentile is apparent (*χ*^2^ = 54.16, *p* < 0.01). McNemar’s pairwise comparisons (with Bonferroni corrections for multiple comparisons) indicated a complex relationship between frequency pairs and is not consistent with an obvious monotonic trend for greater hearing loss at higher audiometric frequency.

**Table 2:** Proportion of individuals falling outside the 95th percentile compared to age normative data for pure-tone audiometry

Test frequency	250 Hz	500 Hz	1 kHz	2 kHz	3 kHz	4 kHz	6 kHz	8 kHz
% of patients outside 95th percentile	78	76	78	63	51	59	81	78
*p* value (chi-squared)	< 0.01	< 0.01	< 0.01	< 0.01	< 0.01	< 0.01	< 0.01	< 0.01

### Tympanometry

Tympanometry results were assessed on an individual ear basis. Results were available for 90 ears. Forty-two (47%) tympanic membranes demonstrated normal type A tympanograms, 10 (11%) were classified as type B, 12 (13%) had negative middle ear pressure (type C) and 4 (5%) had perforations; 22 (24%) of examined tympanic membranes were hypercompliant (type A_d_).

**Fig. 3:**
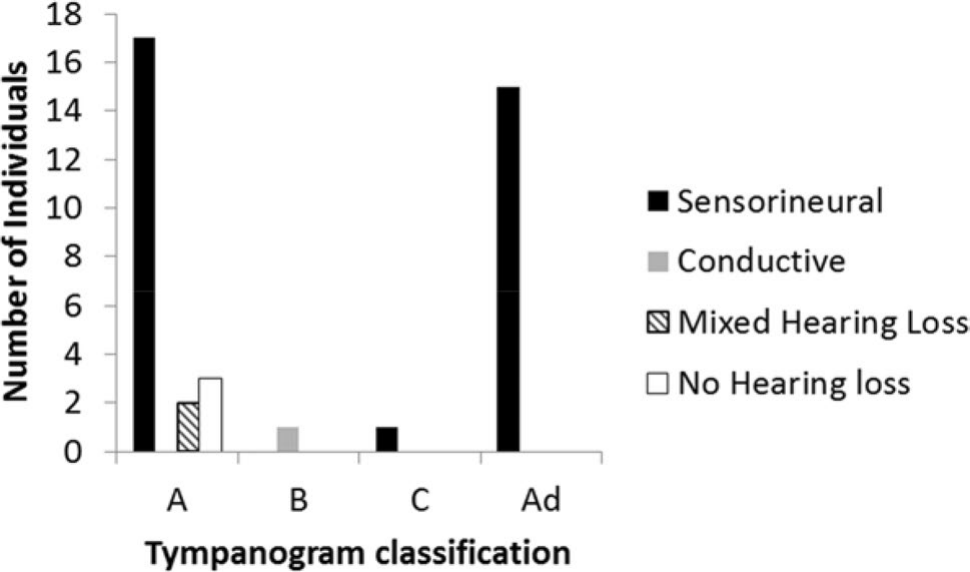
Aetiology of hearing loss according to tympanometry classification

Tympanometry categories correlated to the different aetiologies of hearing loss for 39 ears (measurements for both made on the same day) are shown in Fig. 3. No association with conductive hearing loss is apparent in any of the 15 ears with a Type A_d_ hypercompliant tympanogram.

To assess whether hypercompliant tympanograms were linked to a history of recurrent ear infections, tympanogram outcomes were correlated to the reported otological history of previous ear infection. Only one of the eight patients with a type A_d_ hypercompliant tympanogram who completed the questionnaire reported regular ear infections; of the 18 patients without hypercompliant tympanograms who completed the questionnaire, five patients reported regular ear infections, suggesting ear infections were more common in patients that did not have type A_d_ tympanograms.

### Otological symptoms questionnaire

Forty-five patients completed the otological symptoms questionnaire. Thirty-four (77%) reported some degree of hearing impairment and eight (18%) reported recurrent ear infections, with a mean of three infections a year.

### Speech spatial qualities questionnaire

Table 3 shows mean values for the three categories covered by the Speech, Spatial and Qualities of Hearing Scale questionnaire (Noble and Gatehouse, [21]). The total SSQ score obtained is significantly lower (greater difficulty) than a young, normal-hearing group (unpaired *t* test: *t* = 9.25), but there was no significant difference between the Stickler cohort and a group of individuals with mild–moderate bilateral sensorineural hearing loss (unpaired *t* test: *t* = 0.279, df = 143, *p* = 0.7805). The results from the Stickler type 2 population show that individuals tend to report similar difficulty when discriminating speech, in identifying the properties of sound perception linked to spatial perception, and the quality of sound perception. This is similar to the findings of Demeester et a.l, [22] who tested a young normal-hearing population. The population tested by Gatehouse et al. showed greater difficulty discriminating speech than the other two domains covered by the SSQ, and this may be partially explained by the fact that the population was on average older than the Stickler cohort (70 years (SD 8.3) vs. 37 years (SD 17), respectively).

**Table 3:** SSQ questionnaire results for the three questionnaire domains from three separate cohorts

	Stickler type 2	Gatehouse and Noble [20]	Demeester et al. [21]
Speech	6.1 (2.3)	4.4 (2.4)	8.7 (0.9)
Spatial	6.5 (2.3)	5.6 (2.6)	8.5 (1.2)
Qualities	6.9 (2.0)	6.8 (2.7)	9.3 (0.6)
Total	6.5 (2.2)	5.6 (2.6)	8.8 (0.8)

Stickler type 2, a symmetrical bilateral mild sensorineural hearing population [21] and a young normal-hearing population [22]

## Discussion

Hearing loss in previous Stickler Syndrome studies has been reported to be 80% in type 2 patients [14] and 60% in type 1 patients [16]. However, direct comparison of reported outcomes is difficult due to small cohorts and lack of a clear definition of hearing loss.

In the largest global cohort of genetically confirmed type 2 Stickler Syndrome patients, with explicitly stated hearing loss definitions, we find hearing loss to be common. If audiometric thresholds are averaged across frequencies (four-frequency average) 69% [45/65] of individuals had hearing loss in at least one ear, and the majority of hearing loss was symmetrical. In patients with hearing loss, typically the severity was mild (75% of patients with loss), bilateral (87% of patients with a loss) and sensorineural (77% of ears). The selection of the 4-FA as the metric for hearing impairment was chosen, because it most accurately reflects a hearing loss likely to impair an individual’s ability to follow a conversation. It is relatively conservative as an identifier of hearing loss—for example using a model for age-related sensorineural hearing loss, zero individuals would be expected to have 4-FA greater than 20 dB HL in a population of healthy individuals with only age-related hearing loss of the same size as the cohort examined here. Thus it can be concluded that just over two-thirds of the population tested had hearing loss that can likely be attributed to type 2 Stickler Syndrome. If the least conservative metric for hearing loss is used (any one pure-tone frequency in excess of 20 dB HL), the proportion of individuals with a hearing loss rises to 94%, but this is likely to include individuals with hearing loss attributable to ageing. Furthermore, in keeping with previous studies, audiograms generally displayed a greater impairment in the higher frequency regions [7, 13]. However, when age- and gender-matched normative data are accounted for, hearing loss is present across all test frequencies. This is consistent with widespread cochlear pathology, and this information may be important in future studies to investigate the underlying causes of sensorineural hearing loss in this condition.

Comparison of self-report disability scores obtained from this population and a young normal-hearing group showed the expected increase in reported difficulty, applying to speech discrimination, as well as spatial and sound quality perception [21]. When compared to a non-stickler population with similar average hearing thresholds [11], self-reported handicap scores were not statistically different, inferring that the levels of hearing disability are consistent with pure-tone audiometric thresholds. This would also suggest hearing difficulties are not exacerbated by a significant retrocochlear or processing abnormality, but the observed sensorineural hearing loss is most consistent with impairment in the auditory periphery.

The exact pathophysiology of Stickler Syndrome type 2 associated hearing impairment remains unknown. Temporal bone computed tomography (CT) scans have revealed no macro-deformity of the inner ear [15]. The underlying collagen defect may, therefore, cause hearing loss due to microstructure irregularity. The COL11A1 and COL11A2 proteins have been reported in the developing mouse cochlear [11] and in a mouse model, COL11A1 haploinsufficiency does not cause significant hearing loss [23], whereas in humans haploinsufficiency has been reported to cause mild hearing loss [24]. In the majority of type 2 Stickler Syndrome cases, the pathogenic variant is a missense change or an in-frame deletion [1, 4, 8, 25, 26] and the abnormal COL11A1 pro-alpha chain exerts a dominant negative effect on normal type XI collagen expression in the cochlear extracellular matrix [15]. In rare cases of recessive type 2 Stickler Syndrome due to bi-allelic null or missense/null mutations, no normal pro-alpha 1(XI), is synthesised, resulting in profound hearing loss [27, 28].

24% of patients were found to have hypermobile tympanic membranes; this is lower than the incidence of 36–46% reported in type 1 Stickler Syndrome patients [15, 29]. Otology questionnaire responses suggested that tympanic membrane hypermobility was not acquired through infection-related trauma. It is hypothesised that mutated COL11A1 fibrils are likely to be present in the fibrous layer of the tympanic membrane, where other collagen types have been demonstrated to be present [30]. This motivates further investigation of primary collagen defects present in the tympanic membranes of patients with type 2 Stickler Syndrome and further investigation into the correlation between joint hypermobility and hypercompliant tympanic membranes [15].

A reticence to test below 20 dBHL for a clinical assessment may have potentially introduced testing bias, with the potential for over emphasising differences between the study population and age-matched normative data for mid- and low-frequencies thresholds. However, the existing data suggest the severity of hearing loss in those patients found to have mid- and low-frequency thresholds outside the normal range would not have been affected by this ‘floor’ effect. Thus, low frequency hearing loss reported in the current type 2 Stickler Syndrome population is unlikely to be an artefact, and confirms with previously reported studies [14].

The tympanometry protocol performed in this study utilised probe tones at 226 Hz. Whilst useful clinical information was gleaned, recently developed wideband acoustic immittance techniques describe middle ear function across a wide frequency range, and normative data is now available [31]. Future application of such techniques to individuals with the Stickler Syndromes may yield detailed data about middle ear function.

## Conclusion

This is the largest study investigating audiological phenotype of genetically confirmed type 2 Stickler individuals. The dataset comprises pure-tone audiometry and tympanometry, as well as self-reported handicap and otological pathology information. The study is also the first to consider age–gender matched normative data with regards to hearing impairment in the type 2 Stickler Syndrome. Details are included of how hearing loss aetiology and severity are classified, which is often lacking in previous studies, in addition to strict criteria when defining hearing loss. A high prevalence of cochlear hearing loss, with evidence of dysfunction across the audible frequency range, rather than restricted to the high-frequencies, was demonstrated. Hypermobile tympanograms are likely to be due to intrinsic tympanic membrane defects, rather than associated with previous perforations due to otitis media, but are less common than in type 1 Stickler Syndrome.

## Appendix 1: Otological symptoms questionnaire

Do you feel you have a hearing problem?

Is this in one or both ears?

Does your hearing fluctuate (come and go)?

Do you suffer from regular ear infections?

If so is this just in one ear?

Approximately how many ear infections do you get per year?

Do you get and fluid/infection/pus coming out of your ears?

Have you ever had any operations on your ears (including grommets)?

Do you ever get any ringing or buzzing sounds in your ears that last longer than a few minutes?

Do you ever suffer from a sensation of dizziness that feels like spinning or rotating sensation?
